# How Lazy Are Pet Cats Really? Using Machine Learning and Accelerometry to Get a Glimpse into the Behaviour of Privately Owned Cats in Different Households

**DOI:** 10.3390/s24082623

**Published:** 2024-04-19

**Authors:** Michelle Smit, Rene A. Corner-Thomas, Ina Draganova, Christopher J. Andrews, David G. Thomas

**Affiliations:** School of Agriculture and Environmental, Massey University, Palmerston North 4410, New Zealand; m.smit@massey.ac.nz (M.S.);

**Keywords:** domestic cat, feline, behaviour, home environment, accelerometry, machine learning

## Abstract

Surprisingly little is known about how the home environment influences the behaviour of pet cats. This study aimed to determine how factors in the home environment (e.g., with or without outdoor access, urban vs. rural, presence of a child) and the season influences the daily behaviour of cats. Using accelerometer data and a validated machine learning model, behaviours including being active, eating, grooming, littering, lying, scratching, sitting, and standing were quantified for 28 pet cats. Generalized estimating equation models were used to determine the effects of different environmental conditions. Increasing cat age was negatively correlated with time spent active (*p* < 0.05). Cats with outdoor access (*n* = 18) were less active in winter than in summer (*p* < 0.05), but no differences were observed between seasons for indoor-only (*n* = 10) cats. Cats living in rural areas (*n* = 7) spent more time eating than cats in urban areas (*n* = 21; *p* < 0.05). Cats living in single-cat households (*n* = 12) spent more time lying but less time sitting than cats living in multi-cat households (*n* = 16; *p* < 0.05). Cats in households with at least one child (*n* = 20) spent more time standing in winter (*p* < 0.05), and more time lying but less time sitting in summer compared to cats in households with no children (*n* = 8; *p* < 0.05). This study clearly shows that the home environment has a major impact on cat behaviour.

## 1. Introduction

The domestic cat (*Felis catus*) is one of most popular pets worldwide. In New Zealand, there are over 1.2 million pet cats, of which 74% are considered to be family members by their owners [1]. As every household is unique, the living conditions of pet cats can differ markedly. New Zealand households, for example, often have more than one cat (38%), dogs (10%), and/or children present [1]. In addition to this, cats are kept indoors only (11%), outdoors only (5%), or have both indoor and outdoor access (83%) [1]. The environment a cat is exposed to can therefore be composed of many different conditions that can affect the animal’s welfare [2,3].

The welfare of an animal has been defined as the state of the animal as it attempts to cope with its environment [4]. In New Zealand, animal welfare is assessed using the five domains model, which considers the domains of (1) nutrition, (2) environment, (3) health, (4) behaviour, and (5) mental state [5]. One way to assess the welfare state of an animal is by observing its behaviour. Behaviour has been defined as “the internally coordinated response (actions or inactions) of whole living organisms (individuals or groups) to internal and/or external stimuli” [6]. Thus, behavioural observations can provide valuable information on an animal’s welfare through the over- or under-expression of specific behaviours. To be able to use behavioural observations as an indicator for welfare, however, behaviours need to be measured accurately to understand how they are affected by different stimuli.

Despite humans living with cats for approximately 9500 years [7], little is known about how the home environment influences the welfare and behaviour of pet cats. Having access to the outdoors is generally accepted as beneficial to the cat’s welfare. Indeed, some studies have found that cats kept exclusively indoors show more behaviours that are unacceptable to the owner (i.e., problem behaviours), with twice as much house soiling behaviour reported by the owners [8,9]. A study has reported that cats were more sociable when living in small families without children and had a higher quality of life score, based on care, behaviour, and physical examination, when living with conspecifics [10]. Thus, it is quite clear that many environmental variables have the potential to influence behaviour. However, it is unknown which factors influence which behaviours and how.

A recent review identified that the majority of previous studies focussed on only one variable to ensure that any differences between treatments or groups were most likely the result of that variable [2]. While studies focussing on one variable can be beneficial to identify variables in the environment that might influence the behaviour and welfare of cats, it does not resemble the in-home situation, where the environment is multi-variate. The review also noted that the majority of studies were completed outside the home setting, such as shelters, catteries, or laboratories [2]. A shelter, cattery, or laboratory are unlikely to resemble a home situation, and, therefore, results from these studies are not necessarily transferrable.

It is not surprising that little is known about the effects of a complex environment on cat behaviour. Behavioural studies are very labour-intensive and have traditionally been conducted using observational methods by either scoring the behaviour in real time or from video recordings [11]. When continuously scoring the behaviour of an animal, behaviours can easily be missed, and only one animal per observer can be scored at a time. There is also the risk of observer fatigue if behavioural scoring sessions are long. The majority of studies reviewed by Foreman-Worsley and Farnworth [2] scored behaviour using scan (i.e., instantaneous) sampling, which involves the observer recording the behaviour of an individual animals at predetermined time intervals [12]. With this method, there remains a risk of missing infrequent and/or short-lasting behaviours.

Accelerometers have been shown to have the potential to identify animal behaviour when combined with machine learning techniques (see review by [13]). Watanabe et al. [14] were the first to use accelerometer data from a cat to identify four behaviours with an accuracy > 65%. To date, three other studies have published validated machine learning techniques which identify cat behaviours from accelerometer data. These studies created a machine learning (ML) model with feral cats [15], with pet cats [16], and with colony cats [17]. The model created by [14] used data from only one cat, and the models created by [16] were created from data from a harness-mounted accelerometer. Few pet cats are accustomed to wearing a harness, thus Smit et al. [17] created models for both a collar- and a harness-mounted accelerometer. Creating a model to identify animal behaviour using ML techniques from accelerometer data is labour-intensive, although once a good working model has been created, identifying behaviours is quick and requires little labour.

This study aimed to examine the effects of several environmental conditions (e.g., indoor vs. outdoor, dry vs. mixed diet, and the presence of other cats, dogs, and/or children) and cat-specific conditions (e.g., age group and sex of the cat) on the behaviour of cats. Behaviours were quantified using a previously validated ML model with an overall accuracy of 73% [17]. This study also aimed to examine behavioural differences between summer and winter, and to determine whether the combination of season and environment affected the cats’ different behaviours.

## 2. Materials and Methods

The study was conducted in the Manawatū-Whanganui region, New Zealand. The study was approved by both the Massey University Human Ethics (MUHEC 4000025773) and Animal Ethics Committees (MUAEC 22/24).

### 2.1. Owner and Cat Recruitment

Voluntary response sampling was used to recruit participants and their cat(s) by distributing flyers to local veterinary clinics and throughout Massey University in Palmerston North (Supplementary Materials S1). The flyer referred participants to a questionnaire that included questions about demographics, housing of the cat, and some general information about the cat(s) (Supplementary Materials S2).

A total of 61 cat owners completed the questionnaire, which captured information on 89 cats. Cats were excluded from participation if the questionnaire was incomplete (*n* = 15), if they lived outside the Manawatū-Whanganui region (*n* = 13), or if cats fell outside the age range of 1 to 10 years (*n* = 6). As some health conditions are known to affect behaviour, cats were also excluded if they suffered from a mobility related illness (e.g., osteoarthritis), a urinary tract and/or kidney disease, diabetes, and/or hyperthyroidism (*n* = 4). After excluding cats that did not meet the inclusion criteria (*n* = 29), a total of 60 eligible cats remained. The owners of these 60 cats were invited to participate in the trial. Participation was voluntary, and owners were able to withdraw their cat(s) at any time.

### 2.2. Data Collection

Data were collected over two periods: summer (1 December 2022–28 February 2023) and winter (1 June 2023–31 August 2023). An appointment was organised with each participant, during which their cat(s) were weighed and assigned a body condition score (BCS; 9-point scale) [18] by the same researcher for the whole study. Owners were also asked whether they fed their cats a wet diet, dry diet, or a mix of a wet and dry diet. Participating cats were fitted with a quick release collar, to which an ActiGraph™ wGT3X-BT (ActiGraph™, Pensacola, FL, USA) accelerometer was attached (weighing 19 g and measuring 33 mm × 46 mm × 15 mm). During each sampling period, owners habituated their cats to wearing the collar with the accelerometer over a six-day period (Table 1), followed by seven consecutive days of data collection. At the end of the data collection period, collars and accelerometers were retrieved and raw triaxial acceleration data were downloaded. If a cat lost its collar during the first or second collection period, their data were not included in the analysis.

**Figure 1:**
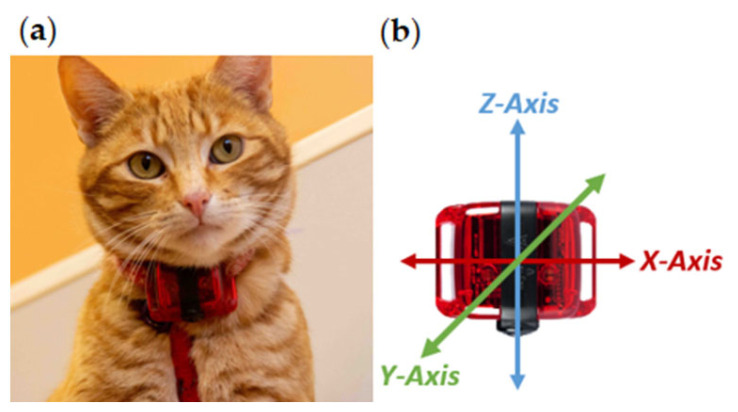
(**a**) Placement and (**b**) orientation of the ActiGraph™ wGT3X-BT accelerometer on a collar.

Accelerometers were positioned ventrally on a collar (Figure 1a), with the orientation of the X, Y, and X axis lateral, dorso-ventral, and cranio-caudal, respectively (Figure 1b). Owners were given instructions on the orientation of the device, to ensure uniform attachment across all participating cats. The ActiGraph™ wGT3X-BT has a dynamic range of ±8 g. Acceleration data were sampled at a frequency of 30 Hz (raw acceleration data), downloaded, and exported into csv files using the ActiLife software (version 6.13.4; ActiGraph ™, Pensacola, FL, USA).

### 2.3. Statistical Analysis

Cat bodyweight and BCS were tested for differences between seasons (summer and winter) using a paired *t*-test and Wilcoxon signed-rank test, respectively, using RStudio v1.4.1 [19]. RStudio was also used to identify the behaviour of cats using the previously validated random forest model which identified eight behaviours: active, lying, sitting, standing, grooming, littering, eating, and scratching [17]. The random forest model required 32 identifier variables to be calculated in order to identify cat behaviours. These identifier variables were derived from the raw acceleration data (30 Hz) and summarized into 1 s epochs (Appendix A). Using the identifier variables and the previously validated random forest model, the behaviours of the cats were identified for each second of the day. For each cat, hourly and weekly proportions of all behaviours were calculated to visualise patterns in behaviour throughout the day.

The frequency of each behaviour was summed on a weekly basis for each cat and merged with data from the questionnaire. The weekly summed frequency of behaviours with variables obtained from the questionnaire was then exported into SPSS (version 29.0.0.0). The effects of different variables and their interactions on the assessed behaviours were tested using generalized estimating equation (GEE) models. GEE models were performed on the total weekly accelerometer count data (seconds) for each behaviour, with individual cats being defined as the subject variable, and season defined as the within-subject variable. It was assumed that the amount of each behaviour displayed by the cat in summer did not affect the amount displayed in winter, and, therefore, the structure of the working correlation matrix was set to independent. As count data were used for the GEE models, a Poisson distribution with log as the link function was selected.

A total of nine variables were included in the analysis: season, sex of the cat, age group, diet, housing, rural vs. urban, multi-cat vs. single-cat household, presence of at least one dog, and the presence of at least one child (<18 years; Table 2). The main effect of all nine variables on each of the eight behaviours were tested (main effects GEE models). A backwards stepwise procedure was followed, removing variables with a *p*-value > 0.10, until only variables remained with *p*-values ≤ 0.10. Given that data were collected for each cat in both summer and winter, the effects of the interaction of season with each of the remaining eight variables for each behaviour were also tested (individual GEE models). For each behaviour, interactions with a *p*-value ≤ 0.10 from the individual GEE models were combined into a multi-interaction GEE model. The same backwards stepwise procedure, as previously used for the main effects GEE, was then followed. For each behaviour, variables that were retained following the backwards stepwise procedure for both the main effects GEE model and multi-interaction GEE model were combined into multivariate GEE models. Another backwards stepwise procedure was followed until only significant variables remained (*p* < 0.05).

## 3. Results

Of the 60 eligible cats identified from the questionnaire, owners of 18 cats did not respond to the invitation to participate in the study, resulting in a total of 42 cats that participated in the first collection period (summer). Of those 42, 5 lost their collars and 2 did not adapt to wearing the monitor and were thus excluded from the trial. In addition, one cat was euthanized due to reasons unrelated to this study between the first and second collection period, one cat was withdrawn from the study by the owner, and two cats were not booked in for the second collection period by the owner. The second collection period therefore included 33 cats, of which 5 cats lost their collar, resulting in a final sample size of 28 cats. Only data from these 28 cats were included in the data analysis.

Cats weighed less in summer (4.6 ± 0.15 kg) than winter (4.8 ± 0.17 kg; *p* = 0.015); however, no difference was found in BCS (median = 6; *p* > 0.05). All cats that participated in the study were desexed; therefore, hereafter they will be referred to as female and male. The cats were fed either a dry diet or a combination of dry and wet diets (e.g., canned or pouched). Of the eligible cats, cats were either housed indoors with unlimited outdoor access (hereafter considered outdoor), or indoors with limited outdoor access (hereafter considered indoor). In one household with two cats participating in the study, a child was born between the first and second collection, which transitioned these cats from a child-free household to a household with a child. In one of the households, a second cat was introduced between the first and second collection period, resulting in a change in classification from a single-cat to a multi-cat household (Table 3).

For each behaviour, the main (Table 4) and interaction effects (Table 5) were modelled, and a backwards stepwise procedure was followed. The main effects and interactions with a *p*-value ≤ 0.10 that were retained following the backwards stepwise procedure were combined into multivariate GEE models, for which the results are shown. Percentages of time spent exhibiting every behaviour are presented as mean ± standard error (SE).

### 3.1. Active

**Figure 2:**
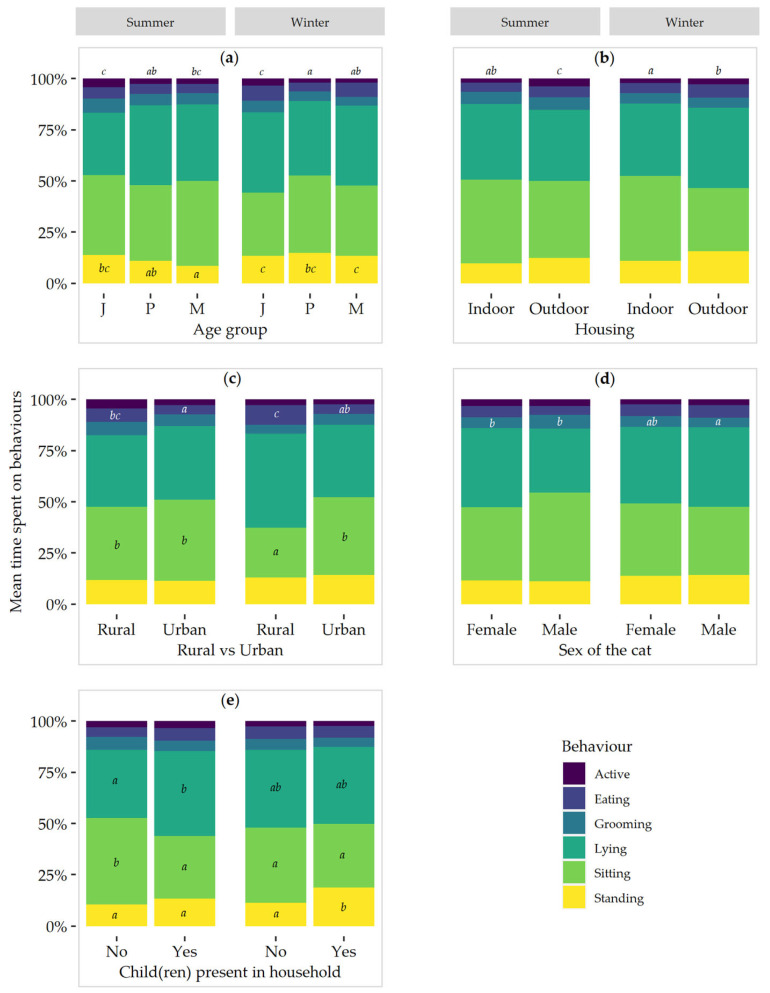
Effect of interaction on the different domestic cat behaviours: (**a**) season × age group with J = Junior, P = Prime, M = Mature; (**b**) season × housing; (**c**) season × ‘rural vs. urban’; (**d**) season × sex of the cat; (**e**) season × child(ren) in household. ^a–c^ Within a behaviour, bars with different superscripts differ significantly (*p* < 0.05). Superscripts above the columns in (**a**,**b**) are for active behaviour.

Overall, cats were active for 2.8 ± 0.25% of their time. There was an interaction of season × age (*p* < 0.001; Figure 2a) and season × housing (*p* < 0.001; Figure 2b) for active behaviour. In summer, junior cats spent more time being active (4.2 ± 0.58%) compared to prime (2.7 ± 0.37%; *p* = 0.008), but not mature cats (2.5 ± 0.73%), while in winter junior cats spent more time being active (3.5 ± 0.41%) than both prime (1.9 ± 0.27%; *p* < 0.001) and mature cats (2.1 ± 0.24%; *p* = 0.002). No seasonal differences were found within age groups (*p* > 0.05).

Cats with outdoor access spent more time active in both summer (*p* < 0.001) and winter (*p* < 0.047) than indoor cats (3.9 ± 0.39% vs. 2.0 ± 0.36% and 2.7 ± 0.33% vs. 2.2 ± 0.23%, respectively). While cats with outdoor access were less active in winter than in summer (*p* = 0.003), there were no seasonal differences in the time spent active for indoor cats (*p* > 0.05).

### 3.2. Eating

Overall, cats spent 5.5 ± 0.46% of their time eating. There was an interaction of season × ‘rural vs. urban’ (*p* < 0.001; Figure 2c), whereby cats living in a rural environment spent more time eating in both summer (*p* < 0.038) and winter (*p* < 0.001) than urban cats (6.4 ± 0.71% vs. 4.6 ± 0.58% and 9.6 ± 1.20% vs. 4.7 ± 0.93%, respectively). Rural cats spent more time eating in winter than summer (*p* < 0.001), but urban cats showed no seasonal differences (*p* > 0.05).

### 3.3. Grooming

Overall, cats spent 5.5 ± 0.30% of their time grooming. There was an effect from age group, with junior cats grooming more than mature cats (6.3 ± 0.49% vs. 5.0 ± 0.38%; *p* = 0.018). A trend was found for the effect of children on the time spent grooming (*p* = 0.096), with cats spending more time grooming in households without children (5.8 ± 0.37%) than those with at least one child (4.6 ± 0.39%).

An interaction effect of season × sex of the cat was found on grooming behaviour (*p* = 0.019; Figure 2d), with male cats spending less time grooming in winter (4.7 ± 0.43%) than summer (6.8 ± 0.86%; *p* = 0.006). No difference was observed between winter and summer for female cats (5.4 ± 0.42% vs. 5.2 ± 0.50%; *p* > 0.05). In addition, within each season, there was no difference in the time spent grooming between male and female cats (*p* > 0.05).

### 3.4. Littering

Overall, cats spent little time littering (0.04 ± 0.01%). There was an interaction of season × age (*p* < 0.001) whereby mature cats spent more time littering in winter (0.06 ± 0.01%) than in summer (0.03 ± 0.01%; *p* = 0.039), and mature cats spent more time littering than junior cats (0.02 ± 0.00%) in winter (*p* < 0.001). No differences in time spent littering were found between summer and winter for junior (0.04 ± 0.02% vs. 0.02 ± 0.00%) and prime cats (0.02 ± 0.01% vs. 0.02 ± 0.00%; *p* > 0.05).

### 3.5. Lying

Overall, cats spent 36.7 ± 1.47% of their time lying. Female cats spent more time lying than male cats (37.9 ± 1.96% vs. 34.9 ± 2.19%; *p* = 0.046). Cats on a dry diet spent less time lying than cats on a mixed diet (34.9 ± 2.50% vs. 38.5 ± 1.49%; *p* = 0.013). Cats in multi-cat households spent less time lying (35.1 ± 1.41%) than cats in single-cat households (39.6 ± 2.82%; *p* = 0.050). A trend was found for ‘rural vs. urban’ on time spent lying (*p* = 0.096), with cats living rurally spending more time lying (40.4 ± 2.76%) than cats living in an urban area (35.5 ± 1.68%).

There was an interaction effect of season × child(ren) in the household (*p* = 0.011; Figure 2e), whereby cats in households where at least one child was present spent more time lying (41.4 ± 1.93%) than cats in households without children (33.2 ± 2.35%; *p* = 0.002) in summer but not winter (38.0 ± 3.25 vs. 37.5 ± 3.71; *p* > 0.05). In addition, no differences in time spent lying were found between seasons for cats in households with or without children (*p* > 0.05).

### 3.6. Scratching

Overall, cats spent very little time scratching themselves (0.12 ± 0.01%). There was an interaction effect of season × ‘rural vs. urban’ (*p* = 0.001), whereby cats that lived rurally spent more time scratching in summer than in winter (0.16 ± 0.04% vs. 0.07 ± 0.02%; *p* = 0.003). In winter, rural cats also spent more time scratching than urban cats (0.07 ± 0.02% vs. 0.12 ± 0.02%; *p* = 0.031). No seasonal differences in time spent scratching were found for cats living in an urban area (summer: 0.13 ± 0.02%, winter: 0.12 ± 0.02%; *p* > 0.05).

### 3.7. Sitting

Overall, cats spent 36.6 ± 2.04% of their time sitting. Cats fed a dry diet spent more time sitting (39.0 ± 3.59%) than cats on a mixed diet (34.2 ± 1.89%; *p* < 0.001). Cats in a multi-cat household spent more time sitting (39.2 ± 2.38%) than cats in a single-cat household (32.4 ± 3.22%; *p* = 0.005). While female cats sat less (35.5 ± 2.90%) than male cats (38.2 ± 2.74%), this was only a trend (*p* = 0.092).

There was an interaction of season × ‘rural vs. urban’ (*p* = 0.048; Figure 2c). Cats living in a rural area spent more time sitting in summer (35.6 ± 3.06%) than in winter (24.3 ± 4.92%; *p* = 0.042). No seasonal difference was found for urban cats (summer: 39.7 ± 2.89%; winter: 38.0 ± 3.97%; *p* > 0.05). In winter, cats living rurally spent less time sitting than cats living in an urban area (*p* = 0.008).

There was also an interaction of season × child(ren) in the household (*p* = 0.006; Figure 2e) whereby cats living in a household without children spent more time sitting in summer (41.9 ± 2.73%) than in winter (36.5 ± 4.34%; *p* = 0.041). No seasonal difference was found for sitting behaviour in households where there was at least one child present (summer: 30.5 ± 2.71%, winter: 31.0 ± 5.45%; *p* > 0.05). In summer, cats in a household without children spent more time sitting than cats in a household with a child(ren) (*p* < 0.001).

### 3.8. Standing

Overall, cats spent 12.7 ± 0.76% of their time standing. There was an interaction of season × age (*p* = 0.002; Figure 2a) whereby in summer, junior cats spent more time standing (13.8 ± 1.84%) than mature cats (8.6 ± 0.64%; *p* < 0.001) and showed a trend of standing more than prime cats (10.9 ± 0.80%; *p* = 0.066). In addition, mature cats spent less time standing in summer than in winter (8.6 ± 0.64% vs. 13.4 ± 1.84%; *p* = 0.001), but there were no differences among the other age groups.

There was also an interaction of season × child(ren) in the household (*p* < 0.001; Figure 2e), whereby cats in households with at least one child spent less time standing in summer (13.4 ± 2.04%) than in winter (18.7 ± 2.59%; *p* = 0.005). However, there was no seasonal difference for households without children (summer: 10.7 ± 0.78%, winter: 11.4 ± 0.74%; *p* > 0.05). In winter, cats in households without children spent less time standing than cats in households with at least one child (*p* < 0.001), but in summer the difference was only a trend (*p* = 0.075).

### 3.9. Daily Pattern of Behaviour

**Figure 3:**
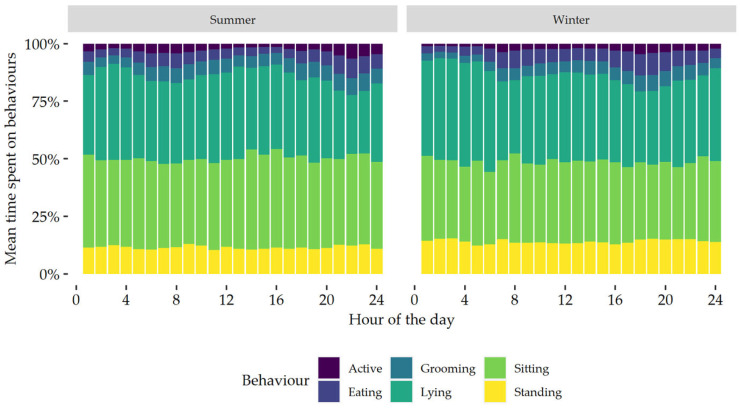
Daily behaviour patterns, expressed per hour, of all the domestic cats in summer and winter.

In both summer and winter, there was a bimodal pattern of behaviour which was driven by active and eating behaviours (Figure 3). In summer, peaks in active and eating behaviours were observed between 05:00–09:00 and 20:00–23:00, while in winter these peaks were between 06:00–08:00 and 16:00–19:00. In summer, between 1 December 2022 and 28 February 2023, the sun rose on 05:21 and 06:57, respectively, and set on 20:50 and 20:02, respectively [22]. In winter, between 1 June 2023 and 31 August 2023, the sun rose on 07:30 and 06:44, respectively, and set on 16:59 and 17:52, respectively [22].

**Figure 4:**
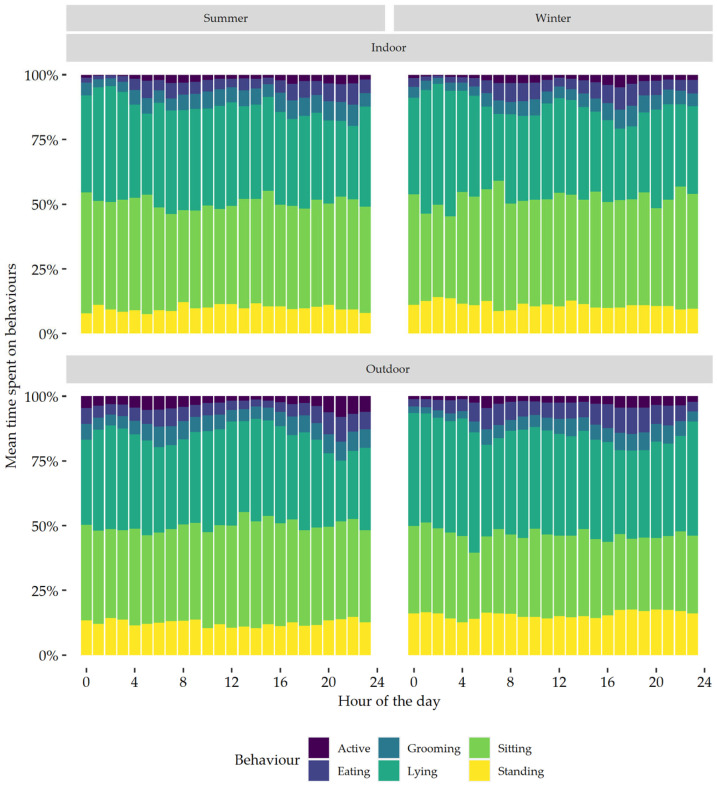
Daily behaviour patterns, expressed per hour, of all the domestic cats in summer and winter living either indoors or having both indoor and outdoor access (outdoor).

**Figure 5:**
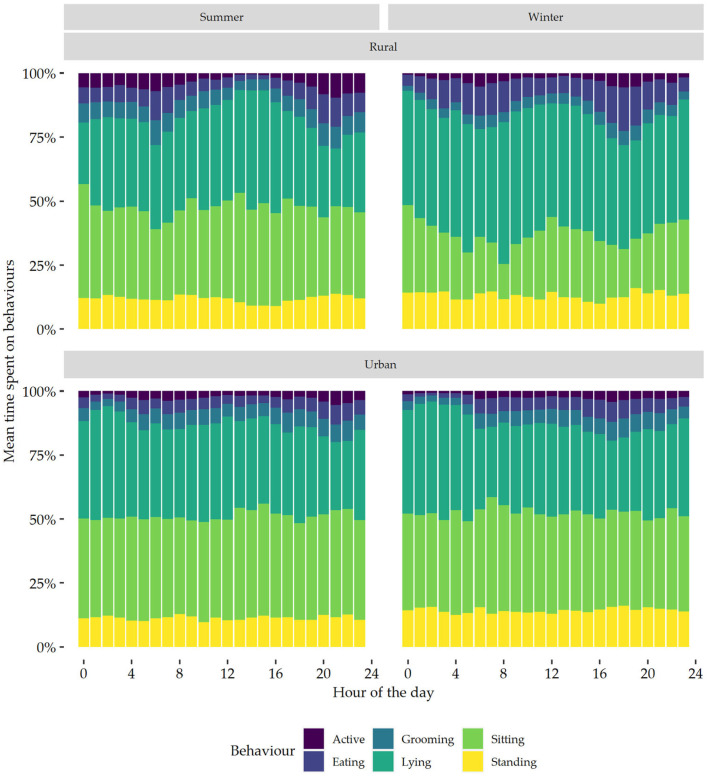
Daily behaviour patterns, expressed per hour, of all the domestic cats in summer and winter living either in rural or urban areas.

Cats which had both indoor and outdoor access showed a clear bimodal pattern of behaviour in both summer and winter; however, the bimodal pattern was less prominent in the summer for cats living indoors (Figure 4). Cats living in rural areas also showed a more pronounced bimodal pattern of behaviour, particularly time spent active and eating, than cats living in urban areas (Figure 5).

## 4. Discussion

The aim of this study was to investigate the behaviour of privately-owned domestic cats and determine how environmental conditions influence their behaviour. Foreman-Worsley and Farnworth [2] identified substantial gaps in our knowledge of how cats respond and interact with their multifactorial home environment. By quantifying cat behaviours, it is possible to determine which behaviours are affected by the home environment and what impact different aspects of this environment have on cats.

Studies on behavioural budgets in domestic cats are limited, and the studies available differ in the methods used to collect behavioural data, the season(s) in which data were collected, and housing conditions. In the current study, housing (indoors only vs. or indoor with outdoor access) showed a significant interaction with the season for active behaviour. As expected, time spent exhibiting active behaviour for indoor cats did not differ between seasons, whereas cats with outdoor access were more active in summer (3.9%) than in winter (2.7%). Two previous studies, both of outdoor colony housed cats, reported an effect of environmental temperature and relative humidity on physical activity [23,24]. Cats in the present study were more active in summer, when temperatures are generally higher than in winter. This was contrary to findings of the outdoor colony housed cats where physical activity declined with increasing temperatures [23,24]. Studies on the effect of weather on cat behaviour are scarce. Cats have a relatively high thermoneutral zone (30–38 °C; [25]), so cats may spend more time indoors in winter, possibly in front of a heat source, where it is warmer. Indoor cats are likely less affected by outdoor weather, as factors such as temperature and relative humidity are less likely to fluctuate indoors. However, a study assessing cat behaviour in response to extreme weather events, that included both indoor only cats (53%) and cats with outdoor access (47%), reported that 75% of cat owners perceived a decline in the level of activity during extreme hot weather events, while 66% of cat owners reported a decline in the level of activity during extreme cold weather events [26]. Not all owners reported an effect of extreme heat or cold on the behaviour of their cat during the study, which could be due to the effect of isolation and the presence or absence of heating and/or cooling sources in the house, affecting temperature fluctuations. It should be noted that owner-based assessment is subjective. Another possible explanation for the higher amount of time spent on active behaviours in summer than in winter by cats with outdoor access is an increase in hunting behaviour in summer. Several studies have shown that cats bring home more prey in summer than in winter [27,28,29], suggesting a higher activity in summer than winter. However, no data on hunting behaviour were collected during this study, so no further conclusions can be drawn. Galea et al. [16], however, successfully identified specific hunting behaviours in domestic cats using accelerometry and hunting behaviours. Thus, accelerometry and machine learning could prove a useful tool for future studies regarding the hunting behaviour of domestic cats.

In the current study, indoor cats were significantly less active than cats with outdoor access in both summer and winter. Berteselli et al. [30] reported that five indoor cats from the same household were twice as active than cats in the current study. It should be noted, however, that an observer was present in the house for behavioural observations in the study by [30], which could have influenced the behaviour of the cats. Smit et al. [24] reported higher physical activity levels when caretakers were present than when they were absent. In addition to this, maintenance behaviours, such as eating and grooming, were included as ‘active’ behaviours by [30], while these were considered separately in the current study. If time spent active, eating and grooming were summed in the current study, cats spent more than twice as much time being “active” than what was reported by [30]. A factor that may contribute to the difference in activity between housing conditions is the area that the cats have access to. It is likely that cats with outdoor access have access to larger areas than cats confined indoors. A study compared activity using accelerometers of cats with access to either 80–100 m^2^ indoors and a 40–80 m^2^ garden (group A), or 200–250 m^2^ indoors and a 2000–2500 m^2^ garden (group B) [31]. In that study, cats from group B were significantly more active than cats from group A. It should be noted, however, that cats in group A were also limited to only one hour per day of garden access, while the cats in group B had free access to the indoors and the garden but were kept in the garden for 11 h during the night [31]. The difference in activity was therefore likely due to a combination of access area and indoor/outdoor access management.

Age has been found to negatively affect overall physical activity in domestic cats [24,32], although it is unclear which behaviours contribute to this decline. Smit et al. [24] reported that cats in the kitten (≤6 months) and junior (>6 months and <3 years) age groups were more active than cats in the prime, mature, and senior age groups (≥3 and <15 years). In the current study, junior cats (≥1 and <3 years) were more active than both prime and mature cats (≥3 and <11 years). The difference between junior and mature cats was not significant in summer, but this was likely due to the large amount of variation observed for mature cats. Grooming and scratching behaviours have been reported to result in high activity counts when measured with accelerometers [33,34]. Indeed, an interaction of season and age on grooming behaviour was found in the present study. Cats in the mature age group spent less time grooming compared to junior cats. When owners were asked about behavioural changes in their older cats (≥11 years of age) compared to when they were younger, they reported a decline in grooming behaviour [35]. The current study suggests that the decrease in overall activity with increasing age reported by [24,32] was likely the result of a decrease in both active and grooming behaviour(s) with increasing age.

The current study found that the absence or presence of a child(ren) and other cats in the household affected some behaviours of cats; however, there was no effect from dogs in the household. Assuming that sitting and standing behaviours are indicative for a greater level of alertness than lying (which also included sleeping), it could be hypothesized that cats in households with at least one other cat or child were more alert. Cats in households have been reported to be less affectionate towards children than adults, especially towards children aged 3–5 years of age [36]. Parents also reported that children were generally the ones seeking an affectionate relationship with the cats, not the other way around [36]. In the current study, cats in multi-cat households spent less time lying and more time sitting than cats in single-cat households, which may also indicate a greater level of alertness. The results were less conclusive for the effect of children in the household. In the winter, cats in households with at least one child spent more time standing than cats in households without children, while in summer, cats in households with at least one child spent more time lying and less time sitting than cats in households without children. In the current study, all cats in households where there was at least one child present had outdoor access. It is therefore possible that in summer, cats spent more time outside, where they were at a lower risk of being disturbed by a child, whereas they may have been indoors more in winter. However, more research is needed to confirm this hypothesis.

When the time spent on each behaviour was plotted on an hourly basis, a bimodal behaviour pattern was observed which consisted of two daily peaks. Based on visual assessment, the bimodal pattern appears to be mainly driven by changes in active and grooming behaviours. A bimodal pattern in activity has previously been reported in domestic cats [24,37,38]. Parker et al. [38] investigated the circadian rhythm of active and eating behaviours of indoor housed cats, using a combination of accelerometry, Ultra-Wide Band tags, and automatic feeding scales, and found a bimodal pattern for both. The peaks in both behaviours predominantly occurred before sunrise and food renewal in the morning, and before sunset in the evening [38]. In the current study, peaks in time spent active and eating also coincided with sunrise and sunset. In the study by [38], ambient humidity and temperature were kept constant, while they were exposed to natural photoperiods through windows. Kappen et al. [37] reported that, under constant temperature, cats exposed to a 16 h photoperiod had a higher activity count than cats exposed to an 8 h photoperiod. Season, temperature, and photoperiod are correlated [39], and it is likely they all affect the behaviour of cats. It should also be noted that pet cats are also exposed to artificial light indoors, especially during the winter when day lengths are shorter. More research is warranted to determine the effects of these factors on feline behaviour.

In the current study, active behaviour was affected by the interaction of season and housing. When visually assessing active behaviour, the bimodal pattern was apparent during both summer and winter for cats with outdoor access, whereas indoor cats showed a bimodal pattern in winter but not summer. It could be hypothesised that the lack of a bimodal pattern of active behaviour of indoor cats in summer may be due to the occurrence of school holidays during this period, which may have resulted in owners and/or family members being home more often. Unfortunately, no data were collected on when owners were at home. Human presence has been shown to affect activity patterns in domestic cats [24,31]. In colony housed cats, ref. [24] reported higher levels of physical activity during hours when staff were present. Similarly, ref. [31] reported that cats with little outdoor garden access (group A) were more active when their owners were at home. Thus, the effect of owner presence on the behaviour of cats warrants further investigation, and recording owner presence should be considered in future studies.

The circadian rhythm of behaviours was also plotted for season × ‘rural vs. urban’. The bimodal pattern, driven by active and grooming behaviours, was visually more pronounced in rural cats than it was in urban cats. Studies comparing the home range of rural and urban cats found that rural cats had much larger home ranges, which could be up to 14.4 times as large [40,41]. A larger home range would likely result in a higher physical activity and more pronounced peaks in activity. The current study, however, did not find an effect of season × ‘rural vs. urban’ on active behaviours. Cats with a larger home range might therefore not be necessarily more active than cats with a smaller home range. The current study did find an interaction of season with ‘rural vs. urban’ on time spent eating, with rural cats spending more time eating than urban cats. This could possibly have contributed to the more pronounced bimodal pattern seen in the rural cats. It is also worth noting that, apart from one cat, all rural cats had outdoor access, whereas the urban cats were composed of both indoor-only cats and cats with outdoor access. The combination of the interaction effect of season × ‘rural vs. urban’ on time spent eating, and the interaction of season × housing on time spent active, could have resulted in the more pronounced bimodal pattern that was visible in the rural cats.

## 5. Conclusions

This study showed that the previously validated machine learning model can be applied to cats in a home situation and that it can be used to determine the effects of environmental variables on cat behaviours. Overall, cats in the current study spent the majority of their time displaying inactive behaviours (lying, sitting, standing: 86%). Cats with both indoor and outdoor access spent more time displaying active behaviours compared to indoor only cats, and were more likely to be affected by weather conditions than indoor-only cats. More research is needed to determine the effect of weather on cat behaviour. The current study further supported the negative relationship between aging and physical activity and found that the decrease in physical activity is most likely driven by a decrease in both active and grooming behaviours. The current study also supported earlier findings of the bimodal pattern of behaviour of cats which appeared to be affected by housing and whether cats lived in a rural or urban areas. More research is warranted in this area, as studies on this topic are currently scarce.

## Figures and Tables

**Table 1:** Training schedule for owners to habituate their cat(s) to wearing the collar with accelerometer, followed by seven days of data collection.

Training Day						
1	2	3	4	5	6	Day 7–14 data collection
2 h	4 h	6 h	8 h	24 h	Off	

**Table 2:** Variables extracted from the questionnaire, or collected during an appointment, and their categories.

Variable	Categories
Season	Summer (December–February) Winter (June–August)
Sex of cat	Entire female Entire male Neutered female Neutered male
Age group [20,21]	Kitten (0–6 months) Junior (7 months–2 years) Prime (3–6 years) Mature (7–10 years) Senior (11–14 years) Geriatric (≥15 years)
Diet	Dry Wet (e.g., canned, pouched, or raw) Mix (mix of dry and wet foods)
Housing	Exclusively indoors Indoors with limited outdoor access (e.g., harnessed walks, catio, or garden access) Indoors with unlimited outdoor access Exclusively outdoors Other
Rural vs. urban	Rural Urban
Multi-cat vs. single-cat household	Multi Single
Presence of at least one dog	No (absent; no dog(s) in household) Yes (present; at least one dog in household)
Presence of at least one child (<18 years)	No (absent; no child(ren) in household) Yes (present; at least one child in household)

**Table 3:** Number of cats for each variable for the summer and winter study periods.

		Season				Season	
		Summer	Winter			Summer	Winter
**Sex**				**Housing**			
	Female	17	17		Indoor ^2^	10	10
	Male	11	11		Outdoor ^2^	18	18
**Age group**				**Number of cats in household**			
	Junior ^1^	10	10		One	12	11
	Prime ^1^	12	12		Two	9	10
	Mature ^1^	6	6		Three	7	7
**Coat length**				**Dogs**			
	Long	5	5		Absent	19	19
	Short	23	23		Present	9	9
**Environment**				**Children (<18 years)**			
	Rural	7	7		Absent	20	18
	Urban	21	21		Present	8	10

^1^ Junior = 1–2 years, prime = 3 to <7 years, mature = 7 to <11 years. ^2^ Indoor = cats with indoor only access, Outdoor = cats having both indoor and outdoor access.

**Table 4:** *p*-values of the interactions from the individual GEE models following a backwards stepwise procedure. Only *p*-values < 0.10 are presented.

	Behaviour							
	Active	Eating	Grooming	Littering	Lying	Scratching	Sitting	Standing
Season × Sex of the cat	NS	NS	0.023	NS	NS	0.079	NS	NS
Season × Age group	<0.001	NS	NS	0.030	NS	NS	NS	0.002
Season × Diet	NS	NS	NS	0.063	NS	NS	0.055	NS
Season × Rural vs. urban	NS	<0.001	NS	NS	NS	0.013	NS	NS
Season × Housing	<0.001	NS	NS	NS	NS	NS	NS	NS
Season × Multi- vs. single-cat household	NS	NS	NS	NS	0.064	NS	NS	NS
Season × Dog(s) in household	NS	NS	NS	NS	NS	NS	NS	NS
Season × Child(ren) in household	NS	NS	NS	NS	0.006	NS	0.0016	<0.001

NS = not significant.

**Table 5:** *p*-values of the variables from the main effects GEE models following a backwards stepwise procedure. Only *p*-values < 0.10 are presented.

	Behaviour							
	Active	Eating	Grooming	Littering	Lying	Scratching	Sitting	Standing
Season	0.006	NS	0.046	NS	NS	NS	NS	0.082
Sex of the cat	NS	0.017	NS	NS	0.049	NS	0.090	NS
Age group	<0.001	0.066	0.057	NS	NS	NS	NS	NS
Diet	NS	0.017	NS	NS	0.015	NS	<0.001	NS
Rural vs. urban	NS	<0.001	NS	NS	0.092	NS	0.027	NS
Housing	<0.001	NS	NS	NS	NS	NS	NS	NS
Multi- vs. single-cat household	NS	NS	NS	NS	0.032	NS	0.006	NS
Dog(s) in household	NS	NS	NS	NS	NS	NS	NS	NS
Child(ren) in household	NS	0.051	0.090	NS	0.005	NS	0.002	0.001

NS = not significant.

## Data Availability

A dataset including the weekly behavioural counts and percentages is available on FigShare at https://doi.org/10.6084/m9.figshare.24848292 (accessed on 18 December 2023) [42].

## Supplementary Materials

The following supporting information can be downloaded at: https://www.mdpi.com/article/10.3390/s24082623/s1, Supplementary Materials S1: Flyer distributed for owner and cat recruitment; Supplementary Materials S2: Questionnaire.

## Appendix A

The random forest model used to identify the behaviours using acceleration data, requires a total of 32 identifier variables. These identifier variables were derived from the raw acceleration data (30 Hz) and summarized into 1 s epochs (i.e., time interval): mean acceleration (X, Y, Z), sum acceleration (X, Y, Z), minimum (min) acceleration (X, Y, Z), maximum (max) acceleration (X, Y, Z), standard deviation (sd) of acceleration (X, Y, Z), skewness (X, Y, Z), kurtosis (X, Y, Z), correlation (XY, XZ, YZ), vector magnitude (VM; mean, sum, min, max, sd, skewness, kurtosis), and overall dynamic body acceleration (ODBA; see Table A1 for detailed description of each identifier variable).

**Table A1:** Description and calculation of identifier variables. Table from [17].

Predictor Variable	Description
Mean	Mean, calculated for every second using the raw acceleration data (30 measures per second)
Sum	Sum, calculated for every second using the raw acceleration data $Sum_{\left(Axis\right)}\sum_{i=1}^{N}Axis_{i}$
Minimum (min)	Minimum value of every 30 measures within each second
Maximum (max)	Maximum value of every 30 measures within each second
Standard deviation (sd)	Measures the spread of the data
Skewness	Asymmetry of the distribution
Kurtosis	Weight of the tails relative to a normal distribution
Vector magnitude (VM)	$VM=\sqrt{X^{2}+Y^{2}+Z^{2}}$
Overall dynamic body acceleration (ODBA)	$ODBA=\sum_{i=1}^{N}\left|DBA_{X}\right|+\left|DBA_{Y}\right|+\left|DBA_{Z}\right|$
Dynamic body acceleration (DBA) ^1^	$DBA=Sum_{axis}-moving\;average$

^1^ Accelerometer data from each axis were individually smoothed using the moving average over 1 s.
