# Supplementary material for: How Lazy Are Pet Cats Really? Using Machine Learning and Accelerometry to Get a Glimpse into the Behaviour of Privately Owned Cats in Different Households
Source: Sensors (Basel). 2024 Apr 19;24(8):2623. doi: 10.3390/s24082623 (PMC11053832; doi:10.3390/s24082623)
Supplement: Supplementary file 1 [file sensors-24-02623-s001.zip › sensors-2804436-supplementary/SM1 - Recruitment flyer.pdf]

Supplementary Material 1 – Flyer distributed for owner and cat recruitment

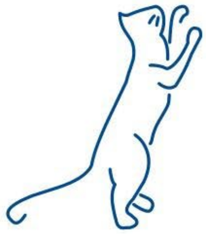

CATS WANTED

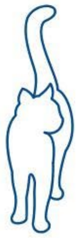

Curious about what your cat does all day?

Enrol your cat in this study and receive a behaviour profile.
Using activity monitors, your cat's behaviour will be monitored for a week in summer and winter. We will be comparing your cat's behaviour between seasons and to other cats living in different housing conditions.

Main criteria

- Housed indoors
- Housed indoors with outdoor access
- Housed outdoors

Other criteria

- Aged between 1 and 10 years
- Used or trainable to wearing a collar or harness
- Available for 2 weeks in summer and winter

[redacted]

For more information and to enrol, please visit the website or scan the QR code.

If you have any questions regarding this study, please send an email to [redacted] with **Cat behaviour study** in the subject line.

This study has been evaluated and approved by the Massey University Animal Ethics Committee. Application ID: AEC 22/24.

This project has been evaluated by peer review and judged to be low risk. Consequently, it has not been reviewed by one of the University's Human Ethics Committees. The researcher Michelle Smit is responsible for the ethical conduct of this research Application ID: 400025773. If you have any concerns about the conduct of this research that you want to raise with someone other than the researcher(s), please contact Professor Craig Johnson, Director (Research Ethics), email humanethics@massey.ac.nz.

MASSEY UNIVERSITY
TE KUNENGA KI PŪREHUROA
UNIVERSITY OF NEW ZEALAND

SCHOOL OF AGRICULTURE AND ENVIRONMENT
