# Supplementary material for: How Lazy Are Pet Cats Really? Using Machine Learning and Accelerometry to Get a Glimpse into the Behaviour of Privately Owned Cats in Different Households
Source: Sensors (Basel). 2024 Apr 19;24(8):2623. doi: 10.3390/s24082623 (PMC11053832; doi:10.3390/s24082623)
Supplement: Supplementary file 1 [file sensors-24-02623-s001.zip › sensors-2804436-supplementary/SM2 - Questionnaire.pdf]

Supplementary Material 2 – Questionnaire

You are being invited to take part in a research study part of a doctoral study. This research will study the behaviour and physical activity of domestic cats in a home environment. To participate in this study, you will need to fill out this questionnaire.

More information on this study can be found in the information leaflet.
The questionnaire will take approximately 5-10 minutes.
*Required

I have read the provided information and agree that research data gathered for this study may be published or made available provided my name or other identifying information is not used *

☐ I agree

I understand that I am free to withdraw from this study at any time *

☐ I agree

Contact information

First and last name *

E-Mail Address *

Phone number *

How would you prefer to be contacted? *
Select one answer

☐ E-mail
☐ Phone

Household information

What is your age category? *
Select one answer

- [ ] < 18 years
- [ ] 18 – 24 years
- [ ] 25 – 34 years
- [ ] 35 – 44 years
- [ ] 45 – 54 years
- [ ] 55 – 64 years
- [ ] > 65 years

How many adults (18 years or older) are present in the household? *
Select one answer

- [ ] 0
- [ ] 1
- [ ] 2
- [ ] 3 or more

How many children (< 18 years) are present in the household? *
Select one answer

- [ ] 0
- [ ] 1
- [ ] 2
- [ ] 3 or more

Please specify the age category or categories of the child(ren) present in the household?
More than one answer possible

- [ ] Baby (up to 1 year)
- [ ] Toddler (1 – 3 years)
- [ ] Preschooler (3 – 5 years)
- [ ] School age (6 – 12 years)
- [ ] Teenager (13 – 18 years)

How many cats are present in the household? *

Select one answer

- [ ] 0
- [ ] 1
- [ ] 2
- [ ] 3
- [ ] 4
- [ ] 5 or more

Are there one or more dogs present in the household? *

Select one answer

- [ ] Yes
- [ ] No

General questions about your cat(s)

If more than one cat is present in your household, please only answer the questions for the cat(s) with who you want to participate in this study. It is possible to fill out part of the following questions for up to 4 cats.

Select the age category for your cat(s) *

Select one answer for each cat

	Kitten (0 - 6 months)	Junior (7 months - 2 years)	Prime (3 - 6 years)	Mature (7 - 10 years)	Senior (11 - 14 years)	Geriatric (15 years or older)
Cat 1	○	○	○	○	○	○
Cat 2	○	○	○	○	○	○
Cat 3	○	○	○	○	○	○
Cat 4	○	○	○	○	○	○

Select the gender for your cat(s) *

Select one answer for each cat

	Entire male	Neutered male	Entire female	Neutered female
Cat 1	◯	◯	◯	◯
Cat 2	◯	◯	◯	◯
Cat 3	◯	◯	◯	◯
Cat 4	◯	◯	◯	◯

Is your cat known with any of the following illnesses? *

Please select the illness(es) that apply to your cat(s)

	Mobility-related illness (e.g. osteoarthritis, fractures)	Urinary tract/kidney disease	Diabetes	Hyperthyroid
Cat 1	☐	☐	☐	☐
Cat 2	☐	☐	☐	☐
Cat 3	☐	☐	☐	☐
Cat 4	☐	☐	☐	☐

Select the living condition of your cat(s) *

Select one answer for each cat

	Exclusively indoors	Indoors with outdoor access	Exclusively outdoors	Other
Cat 1	◯	◯	◯	◯
Cat 2	◯	◯	◯	◯
Cat 3	◯	◯	◯	◯
Cat 4	◯	◯	◯	◯

If you answered "Other" in the previous question, please describe the living condition of your cat(s)

____

____

____

____

____

____

Is your cat used to wearing a collar and/or harness? *

Select one answer for each cat

	Collar	Harness	Collar & harness	Neither
Cat 1	◯	◯	◯	◯
Cat 2	◯	◯	◯	◯
Cat 3	◯	◯	◯	◯
Cat 4	◯	◯	◯	◯

Cat safety collar

To ensure the safety of your cat throughout the study, a cat quick release collar is required while your cat wears the activity monitor. Quick release collars have a type of clasp that pops open easily when force is applied. Below you can see the difference between a quick release collar and traditional collar.

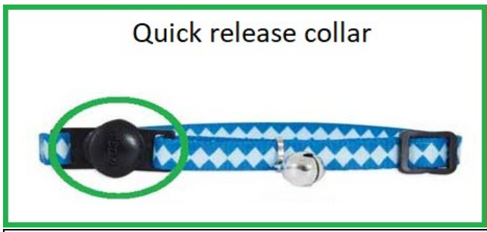

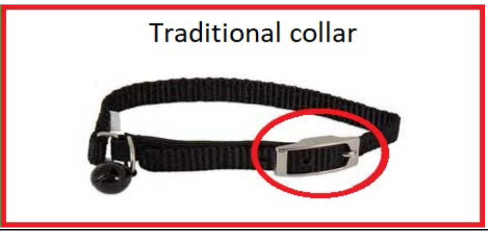

Do you own a cat quick release collar? *

Select one answer

☐ Yes
☐ No

If you do not own a cat quick release collar, one will be provided during the study.

End of questionnaire

You have reached the end of the questionnaire. Thank you for your interest in this study and for completing this questionnaire. Depending on how many people are interested in participating in this study, a selection might be made. You will be informed if you are selected to participate in the study no later than mid November 2022.
